# Racial Disparities in Blood Pressure Control and Treatment Differences in a Medicaid Population, North Carolina, 2005-2006

**Published:** 2011-04-15

**Authors:** Diane Downie, Dorothee Schmid, Marcus G. Plescia, Susan Bostrom, Angie Yow, William W. Lawrence, Sara L. Huston, C. Annette DuBard

**Affiliations:** Public Health Preparedness Program, Division of Public Health. Ms. Downie was affiliated with the North Carolina Department of Health when the research for this article was conducted; North Carolina Department of Health and Human Services, Raleigh, North Carolina; North Carolina Department of Health and Human Services, Raleigh, North Carolina; North Carolina Department of Health and Human Services, Raleigh, North Carolina; North Carolina Department of Health and Human Services, Raleigh, North Carolina; North Carolina Department of Health and Human Services, Raleigh, North Carolina; North Carolina Department of Health and Human Services, Raleigh, North Carolina. Dr. Huston is also affiliated with the University of North Carolina at Chapel Hill, Chapel Hill, North Carolina; North Carolina Department of Health and Human Services, Raleigh, North Carolina. Dr DuBard is also affiliated with the University of North Carolina at Chapel Hill, Chapel Hill, North Carolina

## Abstract

**Introduction:**

Racial disparities in prevalence and control of high blood pressure are well-documented. We studied blood pressure control and interventions received during the course of a year in a sample of black and white Medicaid recipients with high blood pressure and examined patient, provider, and treatment characteristics as potential explanatory factors for racial disparities in blood pressure control.

**Methods:**

We retrospectively reviewed the charts of 2,078 black and 1,436 white North Carolina Medicaid recipients who had high blood pressure managed in primary care practices from July 2005 through June 2006. Documented provider responses to high blood pressure during office visits during the prior year were reviewed.

**Results:**

Blacks were less likely than whites to have blood pressure at goal (43.6% compared with 50.9%, *P* = .001). Blacks above goal were more likely than whites above goal to have been prescribed 4 or more antihypertensive drug classes (24.7% compared with 13.4%, *P* < .001); to have had medication adjusted during the prior year (46.7% compared with 40.4%, *P* = .02); and to have a documented provider response to high blood pressure during office visits (35.7% compared with 30.0% of visits, *P* = .02). Many blacks (28.0%) and whites (34.3%) with blood pressure above goal had fewer than 2 antihypertensive drug classes prescribed.

**Conclusion:**

In this population with Medicaid coverage and access to primary care, blacks were less likely than whites to have their blood pressure controlled. Blacks received more frequent intervention and had greater use of combination antihypertensive therapy. Care patterns observed in the usual management of high blood pressure were not sufficient to achieve treatment goals or eliminate disparities.

## Introduction

Racial and ethnic health disparities have become a prominent issue in the national debate about health care in the United States and have been particularly well-documented in cardiovascular disease (CVD), including stroke, coronary heart disease, heart failure, and high blood pressure (1-3). Death rates from CVD are higher among blacks and have decreased at a slower rate than among whites, effectively widening the disparity (4). High blood pressure is the single most important modifiable risk factor for cardiovascular disease, yet blood pressure control is achieved in only one-third of all patients with high blood pressure (4-8). Among patients with regular medical care, only 48.9% of blacks have their blood pressure adequately controlled, compared with 59.7% of whites (4).

A number of factors are important in achieving adequate control of high blood pressure, including biological, cultural, social, and health care provider and system factors (9).

Although access to health care has dominated the national debate about the inadequacies of the US health care system, racial and ethnic disparities among patients with similar access to care and similar socioeconomic status are known to exist (5). Previous studies have found higher awareness and treatment of high blood pressure among blacks than among whites, but poorer control; demographics, socioeconomic status, comorbidities, and behavioral risk factors appear to play little role in explaining these racial differences (4,6). Among patients receiving care for high blood pressure, provider nonadherence to treatment guidelines or failure to pursue treatment goals aggressively are known to contribute to low attainment of treatment goals for blood pressure. To our knowledge, however, no prior studies have explored the role of clinical practice patterns in racial disparities in blood pressure control.

Medicaid is the largest provider of health insurance for low-income and minority populations in the United States, and Medicaid patients have a disproportionate share of cardiovascular risk factor prevalence, uncontrolled blood pressure, and associated illness and death (10,11). We reviewed the charts of a representative sample of adult Medicaid recipients in North Carolina with diagnosed high blood pressure managed in the primary care setting. The objectives of this analysis were to 1) identify differences in blood pressure control between black and white Medicaid recipients with high blood pressure managed in the primary care setting; 2) examine whether these differences could be explained by differences in demographic factors, comorbidities, or provider characteristics; and 3) determine whether black patients with blood pressure above goal had received differential management for high blood pressure compared with that of white patients during the prior year.

## Methods

### Study population

We used Medicaid administrative data to select a representative sample of North Carolina Medicaid recipients aged 21 years or older with high blood pressure managed in the primary care setting. Recipients were enrolled with Medicaid for at least 11 months from July 1, 2005, through June 30, 2006, and had an office visit with a diagnosis of high blood pressure (ICD9 401xx), excluding pregnancy-induced high blood pressure. We excluded patients who had any office visits with a cardiologist or endocrinologist during this time and those receiving dialysis services for end-stage renal disease. This study was performed as a quality improvement activity of the North Carolina Division of Medical Assistance and was exempted from review by the University of North Carolina Office of Human Research Ethics.

North Carolina had a traditional fee-for-service (FFS) program for Medicaid recipients and 2 managed-care programs during the study period: Carolina ACCESS (CA-I), in which recipients are assigned to a primary care provider (PCP), and ACCESS II (CA-II), which additionally incorporates community-based care management and quality improvement initiatives. PCPs were identified according to administrative assignment for eligible patients in the CA-I and CA-II systems. For FFS patients, the PCP was identified by examining professional services claims submitted during the eligibility year with the following specialty type: general or family medicine, internal medicine, obstetrics and gynecology, pediatrics, federally qualified health center, rural health center, nurse practitioner, or health department. The provider who had submitted the most claims (or the most recent claim in case of a tie) was identified as that patient's PCP. In Medicaid administrative data, "provider" refers to a single physician or a larger practice organization.

To ensure a representative statewide sample and adequate sampling from 8 counties planning a high blood pressure initiative for CA-II enrollees, we used a stratified cluster sampling design and randomly selected PCPs within 4 sampling strata (CA-II patients in pilot counties, CA-II patients in nonpilot counties, CA-I/FFS patients in pilot counties, and CA-I/FFS patients in nonpilot counties). We excluded providers with fewer than 5 eligible patients. A total of 4,046 charts were reviewed from March through July 2007. Of these, we excluded 224 patients from analysis because there was no high blood pressure diagnosis in the chart; 60 patients because they had no office visit after June 30, 2005; and 20 patients because no blood pressure measurement was documented. We limited our analyses to patients identified as black or white in the medical record, or if not available in the record, according to self-reported race in Medicaid enrollment data. We could not determine patient race for 3.5% of charts reviewed. The final sample included data for 2,078 black and 1,436 white patients from a total of 160 providers.

We abstracted medical record data from the offices of selected PCPs by using an electronic clinical abstraction tool developed by Michigan Peer Review Organization and the North Carolina Division of Medical Assistance. Q Mark Inc (Q Mark Inc, Englewood, Colorado) provided trained nurses for the chart abstractions who passed interrater reliability and consistency tests. Reviewers followed systematic guidelines and read all summary documents in the chart as well as clinic notes and correspondence for a 12-month look-back period from the most recent visit. Each chart was reviewed by a single reviewer.

PCP specialty was determined by self-identification of the billing practice as recorded in Medicaid administrative data. Length of time with PCP was calculated on the basis of the earliest service date and the most recent service date documented in the chart. Providers located in a county with a population density of more than 200 people per square mile, according to US Census 2000 data, were classified as urban; all others were classified as rural.

### Measures

All study analyses were based on medical record documentation. The goal for blood pressure treatment was defined as less than 130/80 mm Hg for patients with diabetes and less than 140/90 mm Hg for all others, in accordance with the Seventh Report of the Joint National Committee on Prevention, Detection, Evaluation, and Treatment of High Blood Pressure (JNC 7) (12). A comprehensive, uniform dictionary of all clinical conditions and terms meeting study definitions of high blood pressure, diabetes, hyperlipidemia, cardiovascular disease (including coronary disease, stroke, and peripheral arterial disease), tobacco use, chronic obstructive pulmonary disease, and asthma was used to identify the presence of these conditions as documented in the medical record. Chronic kidney disease was defined as having an estimated glomerular filtration rate (eGFR) <60 mL/min/1.73 m^2^ and was calculated by using the isotope dilution mass spectrometry (IDMS)-traceable Modification of Diet in Renal Disease (MDRD) Study equation from the most recent serum creatinine level documented in the medical record. Body mass index (BMI) was calculated from most recent weight and height documented in the medical record, when available. If no height was recorded in the medical record, the sex-specific median height of the study population was used to calculate BMI. Tobacco use status, creatinine, and weight were not available for 31%, 9%, and 2% of patients, respectively. Antihypertensive agents listed on the patient's medication regimen at the time of abstraction were recorded. Combination therapy was defined as the use of 2 or more of the following antihypertensive drug class categories: angiotensin converting enzyme (ACE) inhibitors, angiotensin receptor blockers, beta blockers, calcium channel blockers, thiazide diuretics, other diuretics, vasodilators, and antiadrenergic agents.

### Statistical methods

We used the most recent blood pressure measurement available from the patient's medical chart to assess the prevalence of above-goal blood pressure in blacks compared with whites. Next, we examined the bivariate relationships between race and patient and provider characteristics that may influence blood pressure control. To assess potential explanations for racial disparities in blood pressure control, we used logistic regression to calculate odds ratios (ORs) for the association between blood pressure control and race (black vs white) and expected covariates. First, in the step 1 full model, we tested for contributions of patient characteristic variables in predicting blood pressure control, including sex, age, comorbidities, and number of medications. Covariates associated with blood pressure control with a *P* value less than .10 were included in the final model. In step 2, we added provider characteristics, including PCP specialty, rural versus urban location, number of years of care with current PCP, and number of visits to PCP during the prior year. Covariates associated with blood pressure control with a *P* value less than .10 were included in the final step 2 model.

To examine the hypothesis that differential treatment patterns may contribute to observed differences in blood pressure control, we analyzed treatment characteristics for the subset of black and white patients with blood pressure above goal. Treatment characteristics included discussion of medication adherence, diet, weight reduction, exercise, sodium restriction, and moderation of alcohol; change in antihypertensive medication regimen in the prior year; and number of antihypertensive drug classes prescribed in combination.

We additionally examined provider response to high blood pressure during office visits within the year before the most recent office visit, up to 5 visits per patient (n = 4,812 visits for blacks, n = 2,931 for whites). For visits with blood pressure above goal, we examined the likelihood that patients had the following care components: 1) documentation of a lifestyle recommendation (any recommendation for medication adherence, diet, weight reduction, exercise, sodium restriction, or moderation of alcohol), 2) change in antihypertensive medication regimen, and 3) a documented plan for follow-up care.

To analyze data, we used SAS versions 9.1 and 9.2 (SAS Institute, Inc, Cary, North Carolina). Weights were applied to correct for the unequal chance of being selected for patient clusters within providers in the 4 sampling strata, and for unit nonresponse. Analyses accounted for the clustering of patients within providers and for stratification. For significance testing, the *F*-adjusted Rao-Scott χ^2^ square and Wald χ^2^ square tests were used.

## Results

The proportion of patients who had met their blood pressure goal was significantly lower among black patients than white patients (43.6% vs 50.9%, *P =* .001) (Table 1). A greater proportion of blacks were women (74% vs 65%, *P <* .01), and age distribution was similar. Although the presence of most comorbidities was similar, blacks were less likely to have hyperlipidemia, chronic kidney disease, chronic obstructive pulmonary disease, asthma, or reactive airway disease, and were less likely to smoke. Whites were more likely than blacks to have 8 or more total active medications (56.6% vs 46.6%). Looking specifically at antihypertensive medications, however, nearly half (46.7%) of blacks were on 3 or more antihypertensive drug classes compared with a third (31.3%) of whites. Geographic location, length of time with current PCP, and number of office visits in the prior year did not differ by race.

In step 1 of the logistic regression modeling (Table 2), when controlling for patient characteristics, blacks were significantly less likely than whites to have their blood pressure controlled (OR = 0.75; 95% confidence interval [CI], 0.61-0.93; *P =* .009). Inclusion of provider characteristics to the model in step 2 had little additional effect on the association between race and blood pressure control (OR = 0.78; 95% CI, 0.64-0.96; *P =* .02), and the relationship remained significant. In addition to race, diabetes, weight status, and PCP specialty other than family practice or internal medicine were associated with poor blood pressure control in the final model.

Among patients who had not achieved their blood pressure goal (n = 1,157 blacks and n = 688 whites) (Table 3), blacks were more likely than whites to have received counseling regarding sodium restriction (12% vs 8.5%, *P =* .006), whereas other types of lifestyle recommendations (medication adherence, diet, weight reduction, exercise, and moderation of alcohol) did not differ significantly by race. Only 47.4% of black and 47.2% of white patients with blood pressure above goal had any documentation of lifestyle recommendations during the prior year. Use of combination antihypertensive therapy was more common among blacks (*P* < .001). Blacks were more likely than whites to have had a change of antihypertensive medication regimen during the prior year (46.7% vs 40.4%, *P *= .02).

A total of 14,583 office visits were reviewed. Blood pressure was elevated during 4,812 (57.2%) office visits during the prior year for blacks, and 2,931 (49.4%) office visits for whites (Table 4). During office visits with above-goal blood pressure, blacks were significantly more likely than whites to have a documented lifestyle recommendation (medication adherence, diet, weight reduction, exercise, sodium restriction, or moderation of alcohol) (17.6% vs 13.9%, *P =* .002) and more likely to have any documented intervention (medication change or lifestyle recommendation) (35.7% vs 30.0%, *P* = .021). There was no significant difference between races in the likelihood of antihypertensive medication change. A follow-up care plan was noted during 64.3% of above-goal visits for blacks and 69.1% of above-goal visits for whites (*P =* .08). Planned follow-up within 4 weeks was noted for only 27% of these visits for both races.

## Discussion

In this statewide sample of Medicaid patients with high blood pressure managed in the primary care setting, blacks were less likely than whites to have their blood pressure controlled. We found that adjusting for observed patient and provider characteristics slightly attenuated the relationship between race and blood pressure control but did not completely explain racial differences.

One strength of this study is that the sample is representative of a statewide Medicaid population with high blood pressure, spanning multiple systems of care and treatment localities. Medicaid recipients are characterized by many factors known to be associated with poor blood pressure control or poor health outcomes, including low socioeconomic status and higher prevalence of multiple comorbidities (13,14). Our findings are consistent with prior observations that racial differences in blood pressure control among treated patients are not explained by socioeconomic factors, nonpharmacological management, health insurance, or comorbidities (3,4,6). Despite health care coverage, access to care, and frequent office visits, an unexplained racial disparity in blood pressure control still exists.

Provider characteristics, and quality and intensity of care have been shown to be significant causes of health disparities (3). Differences in blood pressure control may conceivably be due to less aggressive care patterns in black patients, culturally insensitive care, or other differences in counseling and follow-up (15,16). However, in our study, disparities in blood pressure control do not appear to be explained by differential treatment. Among those with blood pressure above goal, blacks were more likely than whites to have received counseling about sodium intake, to have been prescribed 3 or more blood pressure agents in combination, and to have a change of therapy within the prior year. Within each visit with high blood pressure, the likelihood of medication change and planned follow-up did not differ by race, although blacks were more likely than whites to receive a therapeutic lifestyle recommendation.

Lack of appropriately aggressive care, or clinical inertia, has been cited as a cause for suboptimal control of chronic disease risk factors across much of the US health care system (17). We confirmed considerable evidence of clinical inertia for both black and white patients. Fewer than half of patients with blood pressure above goal had documentation of any lifestyle counseling in the past year. During visits with high blood pressure, medical therapy was changed on only 1 in 5 opportunities. In addition, 28% of black patients and 34% of white patients with uncontrolled blood pressure were treated with fewer than 2 antihypertensive agents, which may not be sufficient to achieve blood pressure goals (18).

We were unable to explore many characteristics of patients, health systems, and environments that may contribute to racial disparities in blood pressure control, including health literacy, medication adherence, and barriers to following therapeutic lifestyle recommendations (19). Racial differences in the metabolic and hormonal pathogenesis of high blood pressure may contribute to the prevalence and severity of high blood pressure among blacks, although differences in socioeconomic conditions, access to care, and health-related knowledge or attitudes are thought to play a larger role (20). Researchers have examined the extent to which perceptions of racial/ethnic discrimination can adversely affect health (21-24). Negative attitudes attributed to discrimination have been linked to adverse physiologic reactions involving blood pressure, and researchers have hypothesized that the chronic triggering of these cardiovascular reactions due to discrimination could lead to the development of high blood pressure (22). These reactions may be caused by various factors, including worry about blood pressure, care-seeking behavior of patients, lack of trust, majority provider behavior toward minority patients, or miscommunication between patients and providers (18,24-27).

This study had several limitations. We may have overestimated blood pressure control in this population because all patients sampled were receiving primary care services, and patients with more complicated disease (those seeing cardiologists and endocrinologists and those on dialysis) were excluded. Our study population had a lower proportion of patients older than 65 years than the source Medicaid population, probably because of these exclusions. Generalizability to other populations is also limited. Medicaid eligibility requires meeting state-specific thresholds of low income and assets, in addition to categorical requirements of being elderly, disabled, or pregnant, or having dependent children. Our analyses were limited to information obtainable in the medical record and relied on the accuracy of clinic blood pressure measurements and completeness of chart documentation, which may be particularly unreliable in assessing the extent of therapeutic lifestyle counseling. We counted as evidence of counseling any mention of lifestyle factors or medication adherence in the visit note.

In summary, the gap between current care and ideal care for both black and white Medicaid recipients with high blood pressure is substantial, even among patients with frequent access to primary care. Racial disparities in blood pressure control are not readily explained by socioeconomic, demographic, or comorbidity differences or by provider characteristics or treatment patterns. Current care patterns are not sufficient to eliminate racial disparities in blood pressure control or to achieve desired treatment goals. The consequences of ineffective health care for high blood pressure, in terms of avoidable cardiovascular illness, death, and health care costs, disproportionately affect blacks. Emerging models of high blood pressure care, incorporating patient-centered care teams and planned, longitudinal stepped care approaches, show promise for improving outcomes across all patient populations (27-30). It cannot be assumed, however, that equal access and equal treatment will lead to equal outcomes. Closing the gap of racial disparities may require a more concerted clinical effort for racial minorities and better coordination between health care providers and community resources that can address cultural and health literacy needs and support patient self-management efforts in the home and community setting. Further research is needed to guide these efforts.

## Figures and Tables

**Table 1:** Blood Pressure Control,^a^ Patient, Provider, and Treatment Characteristics of Medicaid Patients With Hypertension, by Race, North Carolina, 2005-2006^b^

Characteristic	Black (n = 2,078)		White (n = 1,436)		Total (N = 3,514)		*P* Value^c^

	n	Weighted % (95% CI)	n	Weighted % (95% CI)	n	**Weighted % (95% CI)**	
High blood pressure	1,155	54.8 (51.5-58.2)	923	64.4 (60.6-68.1)	2,078	59.2 (56.2-62.1)	<.001
Blood pressure at goal	921	43.6 (40.5-46.8)	748	50.9 (47.1-54.6)	1,669	46.9 (44.2-49.6)	.001
**Age group, y**							
21-39	371	17.8 (15.2-20.5)	242	16.6 (13.4-19.7)	613	17.3 (15.0-19.5)	.68
40-64	1,302	62.3 (59.6-65.1)	917	64.3 (61.3-67.3)	2,219	63.2 (61.0-65.5)	
≥65	405	19.8 (16.4-23.3)	277	19.1 (15.9-22.4)	682	19.5 (16.9-22.2)	
**Sex**							
Men	522	25.8 (23.2-28.3)	490	34.6 (31.9-37.3)	1,012	29.8 (27.7-31.8)	<.001
Women	1,556	74.2 (71.7-76.8)	946	65.4 (62.7-68.1)	2,502	70.2 (68.2-72.3)	
**Weight distribution^d^**							
Normal (BMI <25 kg/m^2^)	298	14.5 (12.5-16.4)	227	15.6 (13.6-17.6)	525	15.0 (13.4-16.5)	.40
Overweight (BMI 25-29.9 kg/m^2^)	441	21.9 (19.3-24.4)	340	23.4 (21.0-25.8)	781	22.6 (20.8-24.3)	
Obese (BMI >30 kg/m^2^)	1,292	63.7 (60.2-67.1)	845	61.0 (58.6-63.5)	2,137	62.5 (60.3-64.6)	
**Comorbidities and risk factors**							
Diabetes	802	39.6 (37.2-42.1)	530	37.9 (34.5-41.2)	1,332	38.8 (36.8-40.9)	.40
Hyperlipidemia	826	41.6 (37.7-45.6)	676	46.7 (43.7-49.7)	1,502	43.9 (41.1-49.3)	.02
Cardiovascular disease	376	19.2 (16.5-21.9)	270	17.4 (14.2-20.7)	646	18.4 (16.2-20.7)	.38
Current tobacco use^e^	564	39.4 (34.7-44.2)	549	51.5 (47.0-55.9)	1,113	45.2 (41.1-49.3)	<.001
Chronic kidney disease (eGFR<60)^f^	412	24.7 (22.0-27.5)	384	28.7 (26.5-30.8)	796	26.5 (24.5-28.5)	.01
COPD or asthma/reactive airway disease	357	17.5 (15.5-19.5)	327	22.8 (19.2-26.4)	684	19.9 (17.9-21.9)	.006
**No. of total active medications**							
0-3	319	14.5 (12.1-16.9)	134	8.8 (6.3-11.3)	453	11.9 (10.3-13.6)	<.001
4-7	811	38.9 (35.5-42.2)	503	34.5 (31.7-37.3)	1,314	36.9 (34.6-39.2)	
≥8	948	46.6 (42.4-50.8)	799	56.6 (53.5-59.8)	1,747	51.2 (5.9-24.3)	
**Provider and treatment characteristics**							
PCP specialty							
General/family practice	971	49.6 (34.9.64.3)	852	63.0 (45.3-80.5)	1,823	55.7 (41.0-70.3)	.03
Internal medicine	821	30.5 (17.4-43.7)	480	27.6 (12.1-43.0)	1,301	29.2 (16.5-41.9)	
Other/unknown	286	19.8 (8.9-30.8)	104	9.5 (1.4-17.5)	390	15.1 (5.9-24.3)	
**Geographic location, by provider county^g^**							
Rural	957	57.3 (43.6-71.0)	666	49.0 (28.9-69.0)	1,623	53.5 (38.2-68.8)	.26
Urban	1,121	42.7 (29.0-56.4)	770	51.0 (31.0-71.1)	1,891	46.5 (31.2-61.8)	
**Length of time with current PCP, y^h^**							
>1	201	8.7 (6.5-10.9)	116	7.6 (5.5-9.7)	317	8.2 (6.6-9.9)	.70
1-2	716	33.1 (27.1-39.1)	476	31.8 (27.0-36.7)	1,192	32.5 (27.5-37.5)	
3-4	360	18.8 (15.1-22.5)	287	20.7 (17.4-24.0)	647	19.7 (16.6-22.7)	
≥5	716	39.4 (32.1-46.7)	535	39.9 (34.7-45.0)	1,251	39.6 (34.1-45.1)	
**No. of PCP visits in past year**							
1-2	262	13.4 (11.1-15.7)	137	9.8 (6.7-13.0)	399	11.8 (9.7-13.8)	.09
3-4	569	26.8 (23.3-30.4)	352	25.9 (22.3-29.4)	921	26.4 (23.4-29.4)	
≥5	1,247	59.7 (54.9-64.6)	947	64.3 (58.4-70.1)	2,194	61.8 (57.4-66.2)	
**No. of antihypertensive drug classes prescribed**							
0-1	722	32.3 (28.8-35.8)	607	40.6 (36.3-44.9)	1,329	36.1 (33.1-39.1)	<.001
2	413	20.9 (28.9-35.8)	370	28.0 (21.4-34.6)	783	24.1 (19.7-28.6)	
3	496	25.5 (23.2-27.9)	276	19.3 (17.6-21.1)	772	22.7 (21.0-24.4)	
≥4	447	21.2 (18.4-24.1)	183	12.0 (9.0-15.0)	630	17.1 (14.3-19.9)	

Abbreviations: CI, confidence interval; BMI, body mass index; GFR, glomerular filtration rate; COPD, chronic obstructive pulmonary disease; PCP, primary care provider; JNC, Joint National Committee on Prevention, Detection, Evaluation, and Treatment of High Blood Pressure.

a Blood pressure at goal according to JNC-7 standards; <130/80 mm Hg for patients with diabetes; otherwise <140/90 mm Hg (12).

b Variables with missing data overall and by race are as follows: tobacco use (overall = 1,077, black = 717, and white = 360), chronic kidney disease (eGF<60) (overall = 332, black = 211, and white = 121), and length of time with current PCP (overall = 107, black = 85, and white = 22). Total n for blacks, 2,078; for whites, 1,436; and overall, 3,514 (no missing data for sex, age group, both blood pressure measures, diabetes, hyperlipidemia, cardiovascular disease, COPD/asthma, provider location, PCP visits in past year).

c *P* value based on *F*-adjusted Rao-Scott χ^2^ testcomparing black with white patients.

d Weight distribution for those patients for whom both height and weight were documented in the medical chart. For patients without height, median height of the population was used (total n = 3,443; black n = 2,031; white n = 1,412. No weight abstracted for 71 patients).

e Tobacco use among those who have been screened for tobacco use and whose status was known (total n = 2,437; black n = 1,361; white n = 1,076).

f Chronic kidney disease for those for whom eGFR was available (total n = 3,182; black n = 1,867; white n = 1,315).

g Providers located in a county with a population density of more than 200 people per square mile, according to US Census 2000 data, were classified as urban; all others were classified as rural.

h For 107 patients, no first visit date was abstracted. Therefore, length of care with their provider could not be established.

**Table 2:** Odds of Blood Pressure at Goal Among Black Versus White Medicaid Patients With Hypertension, North Carolina, 2005-2006

Characteristic^a^	Step 1: Patient Characteristics				Step 2: Treatment Characteristics			

	Full Model		Final Model		Full Model		Final Model	

	Odds Ratio (95% CI)	*P* Value^a^	Odds Ratio (95% CI)	*P* Value^a^	Odds Ratio (95% CI)	*P* Value^a^	Odds Ratio (95% CI)	*P* Value^a^
**Patient **								
Race (black vs white)	0.78 (0.64-0.96)	.02	0.75 (0.61-0.93)	.009	0.79 (0.65-0.97)	.024	0.78 (0.64-0.96)	.02
Age	1.00 (0.99-1.00)	.39	NC	NC	NC	NC	NC	NC
Sex (men vs women)	1.06 (0.82-1.37)	.64	NC	NC	NC	NC	NC	NC
**Weight (vs BMI <25)^b^**								
Overweight (BMI 25-29.9 kg/m^2^)	0.96 (0.75-1.24)	.76	0.88 (0.70-1.11)	.286	0.86 (0.68-1.08)	.202	0.88 (0.70-1.11)	.29
Obese (BMI ≥30 kg/m^2^)	0.75 (0.59-0.97)	.03	0.74 (0.60-0.91)	.005	0.74 (0.60-0.91)	.005	0.76 (0.62-0.93)	.008
**Comorbidities and risk factors**								
Diabetes	0.24 (0.18-0.32)	<.001	0.26 (0.21-0.31)	<.001	0.25 (0.20-0.31)	<.001	0.26 (0.21-0.31)	<.001
Hyperlipidemia	1.00 (0.82-1.21)	.97	NC	NC	NC	NC	NC	NC
Cardiovascular disease	0.86 (0.70-1.05)	.13	NC	NC	NC	NC	NC	NC
Current tobacco use^c^	1.12 (0.91-1.38)	.29	NC	NC	NC	NC	NC	NC
Chronic kidney disease (eGFR <60)^d^	0.86 (0.72-1.04)	.12	NC	NC	NC	NC	NC	NC
COPD or asthma/reactive airway disease	0.96 (0.72-1.28)	.77	NC	NC	NC	NC	NC	NC
No. of medications	1.03 (1.00-1.06)	.03	NC	NC	NC	NC	NC	NC
**Provider and treatment characteristics**								
PCP specialty^e^ (vs family practice)								
Internal medicine Specialty	NC	NC	NC	NC	0.96 (0.73-1.27)	.78	0.96 (0.73-1.26)	.76
Other/unknown specialty	NC	NC	NC	NC	0.68 (0.51-0.90)	.007	0.68 (0.52-0.90)	.007
Rural vs urban^e^	NC	NC	NC	NC	1.06 (0.85-1.32)	.63	NC	NC
**Time with PCP, y^f^**								
<1 (vs >5)	NC	NC	NC	NC	0.89 (0.68-1.17)	.40	NC	NC
1 to <3 (vs >5)	NC	NC	NC	NC	1.00 (0.83-1.21)	.99	NC	NC
3 to <5 of care (vs >5)	NC	NC	NC	NC	1.20 (0.94-1.53)	.14	NC	NC
**No. of PCP visits**								
1-2 (vs >5)	NC	NC	NC	NC	0.74 (0.52-1.06)	.10	NC	NC
3-4 (vs >5)	NC	NC	NC	NC	0.91 (0.76-1.10)	.32	NC	NC

Abbreviations: CI, confidence interval; NC, not calculated; BMI, body mass index; COPD, chronic obstructive pulmonary disease; PCP, primary care provider; GFR, glomerular filtration rate.

a Calculated with Wald χ^2^ test.

b BMI is calculated as weight in kilograms divided by height in meters squared.

c Tobacco use among those who have been screened for tobacco use and whose status was known (total, n = 2,437; black, n = 1,361; white, n = 1,076).

d Chronic kidney disease for those for whom eGFR was available (total, n = 3,182; black, n = 1,867; white, n = 1,315).

e Providers located in a county with a population density of more than 200 persons per square mile, according to US Census 2000 data, were classified as urban; all others were classified as rural.

f For 107 patients, no first visit date was abstracted. Therefore, length of care period with their provider could not be established.

**Table 3:** Treatment Characteristics for Medicaid Patients at Above Goal Blood Pressure,^a^ by Race, North Carolina, 2005-2006

Treatment Characteristic	Black (n = 1,157)		White (n = 688)		*P* Value^b^

	n	Weighted % (95% CI)	n	Weighted % (95% CI)	
**PCP discussed the following topics during the year**					
Medication adherence	159	15.0 (11.8-18.2)	76	12.1 (7.7-16.6)	.22
Diet	316	28.6 (22.9-34.3)	210	30.6 (23.3-38.0)	.53
Weight reduction	160	13.8 (9.90-17.7)	116	16.7 (13.1-20.2)	.18
Exercise	243	22.6 (17.3-27.9)	140	21.8 (17.2-26.3)	.70
Sodium restriction	132	12.0 (8.3-15.7)	67	8.5 (5.9-11.1)	.006
Moderation of alcohol	35	2.6 (0.3-5.0)	20	2.0 (0.0-4.0)	.42
**Any lifestyle recommendation was provided^c^**					
No	640	52.6 (46.4-58.8)	375	52.8 (46.6-58.7)	.97
Yes	517	47.4 (41.2-53.6)	313	47.2 (41.3-53.2)	
**Number of antihypertensive drug classes prescribed**					
0-1	352	28.0 (24.4-31.5)	245	34.3 (27.1-41.6)	<.001
2	233	20.8 (18.1-23.4)	193	30.9 (22.5-39.3)	
3	287	26.6 (23.8-29.4)	150	21.3 (18.3-24.4)	
≥4	285	24.7 (21.6-27.7)	100	13.4 (9.6-17.2)	
Change in antihypertensive medication regimen in the prior year	552	46.7 (41.9-51.5)	283	40.4 (35.6-45.2)	.02
**Screened for the following risk factors**					
Diabetes	1,106	96.3 (94.8-97.9)	648	95.0 (92.8-97.2)	.28
Cholesterol	943	82.5 (79.0-85.9)	586	85.2 (81.1-89.3)	.30
Family history	583	55.1 (46.2-64.0)	421	65.2 (55.7-74.7)	.04
Smoking	764	68.7 (61.0-76.4)	511	74.0 (65.4-82.7)	.28
Obesity	275	22.3 (15.4-29.2)	182	27.8 (21.6-34.0)	.07

Abbreviation: CI, confidence interval; PCP, primary care provider.

a High blood pressure was defined as ≥140/90 mm Hg and ≥130/80 mm Hg for patients with diabetes (12).

b Calculated with *F*-adjusted Rao-Scott χ^2^ test.

c Includes any documentation that medication adherence, diet, weight reduction, exercise, sodium restriction, or moderation of alcohol was addressed.

**Table 4:** Provider Response to High Blood Pressure^a^ During Office Visits, by Race, North Carolina, 2005-2006

Provider Response	Office Visits With Black Patients With High Blood Pressure, n = 4,812 (57.2%)		Office Visits With White Patients With High Blood Pressure, n = 2,931 (49.4%)		*P* Value^b^

	n	**Weighted % (95% CI)**	n	**Weighted % (95% CI)**	
Change in antihypertensive medication regimen	1,064	22.5 (18.9-26.1)	553	19.6 (16.3-22.9)	.18
Lifestyle recommendation (total)^c^	843	17.6 (14.5-20.6)	413	13.9 (11.5-16.2)	.002
Visits during which any intervention was noted (medication or lifestyle recommendation)	1,683	35.7 (30.9-40.4)	864	30.0 (26.5-33.6)	.02
**Any plan for follow-up**					
Yes	3,051	64.3 (56.9-71.6)	1,979	69.1 (64.5-73.7)	.08
No	1,761	35.7 (28.4-43.1)	952	30.9 (26.3-35.5)	
**Follow-up plan within 4 weeks**					
Yes	1,298	27.0 (23.7-30.2)	802	27.1 (23.4-30.8)	.95
No	3,514	73.0 (69.8-76.3)	2,129	72.9 (69.2-76.6)	

Abbreviation: CI, confidence interval.

a High blood pressure was defined as ≥140/90 mm Hg and ≥130/80 mm Hg for patients with diabetes (12).

b Calculated with *F*-adjusted Rao-Scott χ^2^ test.

c Includes any documentation that medication adherence, diet, weight reduction, exercise, sodium restriction, or moderation of alcohol was addressed.
